# Genetic Profile of African Swine Fever Viruses Circulating at Pig Farms in South Korea during the Outbreaks between 2022 and April 2023

**DOI:** 10.3390/v15071552

**Published:** 2023-07-14

**Authors:** Ki-Hyun Cho, Dae-Sung Yoo, Seong-Keun Hong, Da-Young Kim, Min-Kyung Jang, Hae-Eun Kang, Yeon-Hee Kim

**Affiliations:** 1 Foreign Animal Disease Division, Animal and Plant Quarantine Agency, Gimcheon 39660, Republic of Korea; vet10@korea.kr (K.-H.C.); hongsky@korea.kr (S.-K.H.); kdy04207@naver.com (D.-Y.K.); mkjang0506@korea.kr (M.-K.J.); kanghe@korea.kr (H.-E.K.); 2 College of Veterinary Medicine, Chonnam National University, Gwangju 61186, Republic of Korea; shanuar@jnu.ac.kr

**Keywords:** African swine fever virus, variations, pig farm, South Korea

## Abstract

Fifteen pig farms were affected by African swine fever (ASF) in South Korea during the outbreaks between 2022 and April 2023. The ASF virus (ASFV) genome was directly extracted from the blood and tissue samples of 15 ASFV-positive pig farms to analyze the genetic characteristics. Phylogenetic analysis revealed that the 15 strains belonged to p72 genotype II and CD2v serogroup 8, which were the central variable region (CVR) I variants of the *B602L* gene. Fourteen strains were intergenic region (IGR) II variants, containing an additional tandem repeat sequence (TRS), between *I73L* and *I329R*, with the exception of one strain from an ASFV-infected pig farm reported on 22 January 2023, which was an IGR I variant. In addition, a single-nucleotide polymorphism (SNP) was detected at position 107 from the start of the IGR between *A179L* and *A137R* in six isolates. The findings of this study suggest that the sources of the virus at the pig farms from which these variants originated differed from those of other pig farms.

## 1. Introduction

African swine fever (ASF), an infectious disease affecting swine species, is characterized by clinical signs such as acute hemorrhagic fever and death. Lethality induced by highly virulent ASF virus (ASFV) strains can reach up to 100%. ASFV is a large double-stranded DNA virus belonging to the family *Asfarviridae* and genus *Asfivirus* [1]. The size of the ASFV genome ranges from 170 to 190 kb [1,2]. The first ASF case was described in Kenya in 1921 [3]. ASF has been endemic in sub-Saharan African countries for more than 100 years and transmitted to countries beyond Africa, causing two major epidemics. The first epidemic, of which the causative ASFV was p72 genotype I, began in Portugal in 1960 and lasted in the Iberian Peninsula until 1999, causing disseminations to other European, South American, and Caribbean countries [4]. Since its emergence in Georgia in 2007, the second epidemic has been spreading throughout Europe and Asia. In Asia, China was the first country to report an ASF outbreak at a pig farm in Liaoning Province in August 2018 [5]. Thereafter, the disease spread rapidly within China. According to reports by the World Organization for Animal Health (WOAH), ASF had emerged in many Asian countries, including Mongolia, Vietnam, Cambodia, North Korea, Laos, Philippines, Myanmar, and Timor Leste, by 2019.

South Korea reported its first case of ASF at a pig farm in Paju City on 16 September 2019. By 9 October 2019, 14 cases had been detected in the northwestern border region. The most affected administrative districts were Paju City (five cases) and Ganghwa County (five cases), followed by Gimpo City (two cases) and Yeoncheon County (two cases) [6]. The proactive control measures taken by veterinary authorities, including the pre-emptive slaughtering and culling of all pigs on farms in the affected regions and the prohibition of swill feeding in pig farms nationwide, contributed to reducing the number of ASF outbreaks and decreased the possibility of further transmission to other regions [7].

The first case of an ASFV-infected wild boar was reported on 2 October 2019, near the demilitarized zone (DMZ) in Yeoncheon County [8]. ASFV in wild boar populations has been continuously expanding southeastward along the mountain ranges in South Korea. In response, authorities have implemented the massive surveillance of wild boars, density reductions by hunting and trapping, the search and disposal of carcasses, and fencing [9]. Nevertheless, as of 30 April 2023, 3097 cases of ASFV-infected wild boars had been reported in 34 cities/counties. The risk of spillover from infected wild boars to domestic pig farms has continued to increase. The swine industry occupies approximately 30% of the livestock industry in South Korea. It is a highly intensive section with 11,110,726 pigs distributed to 5822 farms as of March 2023 [10]. The portion of backyard pig farms is extremely low. Historically, the Korean pig industry has been affected by epidemics of infectious diseases, such as classical swine fever, foot-and-mouth disease, and ASF, which suggests a need to improve the biosecurity levels of farms in the country. In October 2020, two cases of ASFV were confirmed on pig premises in Hwacheon County, located in the north-central border region [11]. In 2021, five intermittent outbreaks were reported on pig farms in Gangwon Province [12]. Between 2022 and April 2023, 15 ASFV infections have been confirmed on 15 pig farms in Gangwon Province and in the northwestern border region of Gyeonggi Province, where cases of ASF had not been reported since October 2019. Four outbreaks occurred in the Gangwon Province between May and September 2022. On 28 September 2022, one case each was reported in Gimpo City and Paju City. In January 2023, ASFV infection was detected on one pig farm in Pocheon and Gimpo City, followed by one case reported in Yangyang County in February 2023. In March and April 2023, four cases of ASF were reported in Pocheon City, where the pig density was the highest within the northern border region.

Although ASF is highly lethal and has major socioeconomic consequences in affected countries, no effective vaccines or treatments are available for the control of the disease. Elucidating the source of infection and the transmission patterns, and detecting ASF during the initial phase, are of paramount importance in controlling the disease efficiently. Analysis of the polymorphic genome sequences of ASFV can help to reveal the sources of introduction and the features of disease transmission. The mutation rate of the ASFV genome is relatively low. Nonetheless, multiple genomic sites have exhibited polymorphisms. The ASFV strains circulating worldwide have been classified into 24 genotypes based on the differences in the variable region of the p72 gene (*B646L*) [13,14]. ASFV can be subtyped using sequence information from CD2v (*EP402R*), the central variable region (CVR) of the *B602L* gene, and the tandem repeat sequence (TRS) between the *I73R* and *I329L* genes. An analysis of other genes, such as *O174L*, *K145R*, *Multigene family (MGF) 505-5R*, the *CP204L* encoding p30 protein, the *Bt/Sj* region, the *J268L* region, the intergenic regions between the *A179L* and *A137R* genes and between the *MGF 505 9R* and *10R* regions, and the ECO2 region consisting of the IGR between the *I329L* and *I215L* genes and partial *I215L*, was conducted to differentiate between closely related ASFV strains [15,16].

A previous study analyzed 12 genes of 21 ASFV strains (1st–21st infected farms) isolated between 2019 and 2021 [12]. In the present study, we conducted the genetic characterization of 15 ASFV strains isolated from pig farms (22nd–36th infected farms) between 2022 and April 2023. The target genes, including the following 12 gene markers, were analyzed to monitor the potential emergence of new viral variants at the farm level and help to trace the possible sources of the virus: p72 genotype; CD2v serogroup; CVR region of the *B602L* gene; and IGR variations between the *I73R* and *I329L*, *A179L* and *A173R*, and *MGF 505 9R* and *10R* genes; *O174L*; *K145R*; *MGF 505-5R*; *CP204L*; the *Bt/Sj* region; the *J268L* region; and a new potential gene that was recently identified, which was the ECO2 region. The ECO2 regions of 21 ASFVs obtained from 2019 to 2021 were also analyzed.

## 2. Materials and Methods

### 2.1. Sample Collection and Laboratory Detection of ASFV

The ASF Standing Operating Procedure (SOP), a notification established by the Ministry of Agriculture, Food, and Rural Affairs (MAFRA) of South Korea, is a contingency plan that designates stipulated methods, including diagnosis, control measures, and follow-up management, to be executed routinely in designated situations. The National Surveillance System, which was also established by the MAFRA, clarifies the subjects of sampling, testing, genetic analysis, and virus isolation. Accordingly, provincial veterinary services with jurisdiction collected samples, including tissue or EDTA-treated whole blood, from 15 pig farms and confirmed them from 2022 to April 2023. All the ASFV-positive samples were transferred to the National Reference Laboratory of ASF at the Animal and Plant Quarantine Agency (APQA) for genetic analysis and viral isolation.

Viral nucleic acids were directly extracted from homogenized organ samples and whole-blood samples using the Maxwell^®^ RSC Total Nucleic Acid Kit and Maxwell^®^ RSC Whole-Blood Extraction Kit (Promega, Madison, WI, USA). The WOAH TaqMan^®^ real-time PCR, using the primer set composed of primer 1 (5′-CTG-CTC-ATG-GTA-TCA-ATC-TTA-TCG-A-3′) and primer 2 (5′-GAT-ACC-ACA-AGA-TC(AG)-GCC-GT-3′) and probe (5′-FAM-CCA-CGG-GAG-GAA-TAC-CAA-CCC-AGT-G-3′-TAMRA), as described previously [17], was performed using the assays on the CFX96 instrument (Bio-Rad Laboratories, Hercules, CA, USA) to confirm whether the tested samples were positive.

### 2.2. PCR Assay

Thirteen gene markers were amplified and sequenced to genetically characterize the additional 15 ASFV strains. The *B646L* gene encoding p72 for genotyping and the *EP402R* gene for serogroup classification were amplified as previously reported [18,19]. To discriminate between the Korean ASFV strains, PCR primers and previously reported conditions were used to amplify the CVR in the *B602L* gene [18], the IGR between *I73R* and *I329L* (IGR_I73R-I329L_) [20], the IGRs between *A179L* and *A137R* (IGR_A179L-A137R_) [21] and between *MGF 505 9R* and *10R* (IGR_MGF 505 9R/10R_) [22,23], *CP204L* [24], *O174L*, *K145R*, *MGF 505 5R* [25], the *Bt/Sj* region, the *J268L* region [26], and the ECO2 region [16]. The PCR products were sequenced by Macrogen (Daejeon, Republic of Korea).

### 2.3. Genetic Analysis of ASFV Strains

Alignment of all sequences was performed using BioEdit 7.2 (Ibis Biosciences, https://www.mbio.ncsu.edu/bioedit/bioedit.html accessed on 8 May 2023). Phylogenetic analyses were performed using MEGA 11 (MEGA Software, https://www.megasoftware.net accessed on 12 May 2023). A neighbor-joining (NJ) tree for the partial *B646L* sequences was constructed using the maximum likelihood method. The Kimura 2-parameter model was used to construct a phylogenetic tree of the *EP402R* gene.

## 3. Results and Discussion

**Figure 1:**
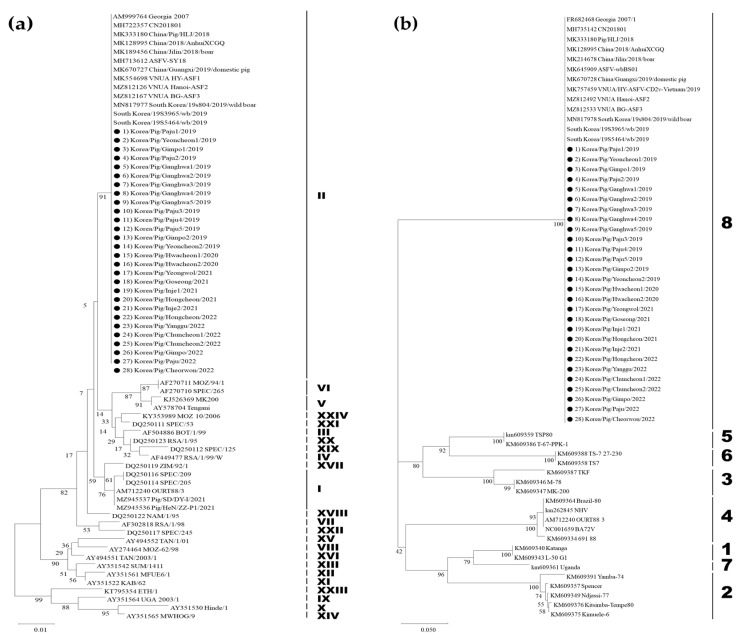
Phylogenetic trees of the 15 African swine fever virus (ASFV) strains isolated from pig farms between 2022 and April 2023 based on partial sequencing of the *B646L* gene encoding p72 and the *EP402R* gene encoding CD2v. (**a**) p72 genotype and (**b**) CD2v serogroup. The neighbor-joining method and Kimura 2-parameter model were used to construct both phylogenetic trees in MEGA 11. The black circles represent the 15 Korean ASFV strains isolated from pig farms between 2022 and April 2023.

The partial *B646L* and *EP402R* sequences of the 15 ASFV isolates (22nd–36th isolates) analyzed in this study were 100% identical to the corresponding regions of Georgia 2007/1 (GenBank Accession No.: NC044959) and the first Korean ASFV isolate designated as Korea/Pig/Paju1/2019 (GenBank Accession No.: MT748042). All 15 ASFV strains belonged to p72 genotype II and CD2v serogroup 8 (Figure 1). p72 genotype II is the most common genotype worldwide. Although p72 genotype I ASFV strains without hemadsorption capacity were identified in China in 2021 [27], which resulted from the use of illegal vaccines [28], p72 genotype II remains the most prevalent in Eurasian countries. Most p72 genotype II ASFV isolates were classified as CD2v serogroup 8. The CVR regions in the *B602L* genes of the 15 ASFV strains were also 100% homologous to those of Georgia 2007/1 and Korea/Pig/Paju1/2019, which correspond to genotype II variant I (CVR1, Georgia variant type). Fourteen ASFV strains from the pig farms in South Korea were IGR_I73R-I329R_ II variants containing an additional TRS, with the exception of the ASFV strain from an ASFV-infected pig farm in Gimpo City, which was reported on 22 January 2023. The ASFV isolate was an IGR I variant (GenBank Accession No.: OQ417675). To our knowledge, this is the first time that the IGR I variant has been identified on a pig farm in South Korea. Table 1 summarizes the results of the genetic analysis of 15 ASFV strains (22nd–36rd) isolated from pig farms between 2022 and April 2023, with 21 ASFVs (1st–21st) previously isolated between 2019 and 2021.

Ten other genes were analyzed to genetically discriminate between the 15 ASFV strains. The CVR region in the *B602L* gene may be an option for intragenotypic clustering. The 15 Korean ASFV strains were CVR1 variants containing 10 tandem amino acid repeat sequences (BNDBNDBNAA) that were 100% identical to the corresponding regions of Georgia 2007/1, Pig/HLJ/2018, China/2018/AnhuiXCGQ, and VNUA/HY-ASF1. CVR1 with single-nucleotide polymorphisms (SNP) 1, 2, and 3 has been detected in Estonia, Poland, and Lithuania. CVR2 without three amino acid tandem repeats has been reported in Estonia [29]. However, the CVR1-SNP and CVR2 variants have not been reported in Asian countries.

**Figure 2:**
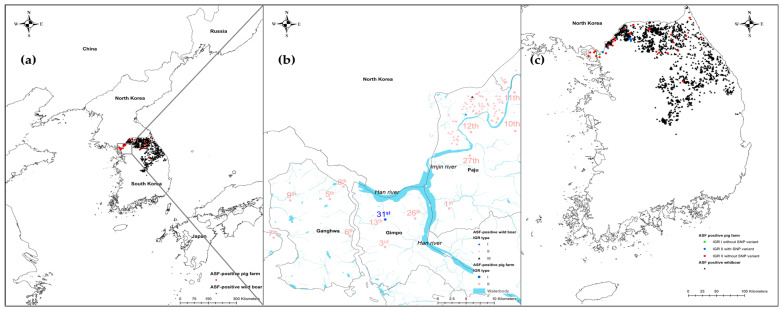
Regional and locality maps showing African swine fever virus (ASFV)-positive pig farms and wild boars in South Korea between 2019 and April 2023. (**a**) The 36 pig farms (red circles) and 3097 wild boars (black triangles) confirmed in South Korea during this period, (**b**) locality of 31st pig farm identified with intergenic region (IGR) between the *I73R* and *I329L* I variant (blue circle) and wild boars infected with IGR I and III variants (blue and black triangles, respectively), and (**c**) ASFV strains with single-nucleotide polymorphisms (SNPs) in IGR between *A179L* and *A137R* at position 55404 in Georgia 2007/1.

IGR_I73R-I329L_ is an important genetic marker for discrimination between p72 genotype II viruses. The insertion and deletion of the TRS in the IGR are largely caused by slipped-strand mispairing during DNA replication [30]. IGR_I73R-I329L_ has been used to trace the source of ASFV and to analyze the spread of the disease in Eastern Europe [20,25]. IGR_I73R-I329L_ contains 10 nucleotides of TRS “TATATAGGAA.” Four IGR variants were classified according to the repetition number of TRS: IGR I (two copies), IGR II (three copies), IGR III (four copies), and IGR IV (five copies) [20]. The first ASFV strain isolated from pig farms and wild boars in South Korea was the IGR II variant [6,8]. This was the first time that the IGR II variant had been detected in a pig farm in the country. In contrast, IGR I (Korea/19S3965/wb/2019) and III (Korea/19S5464/wb/2019) had already been detected in wild boars found near the DMZ in Paju City in December 2019 [31]. The localities of the pig farm and wild boars identified with IGR I and III variants are indicated in Figure 2b. Various IGR variants have been identified in Europe and Asia. IGR I and II have been identified in Russia since 2008 [32]. IGR II is the most common genotype in Europe and Asia. The IGR I variant was detected in an ASFV strain from a wild boar on 18 November 2018, in Jilin Province, adjacent to North Korea [33]. Subsequently, the IGR III variant was detected at a pig farm in the southern region of China on 7 March 2019 [34]. Between 2019 and 2021, the IGR II variant was prevalent in pig farms in Vietnam. However, IGR I, III, and IV were detected in pig premises between 2019 and 2021 [35,36]. IGR I, III, and IV were detected between 2017 and 2020 in Poland [16,25]. Information on the IGR variants identified in Europe and Asia is presented in Table 2.

**Figure 3:**
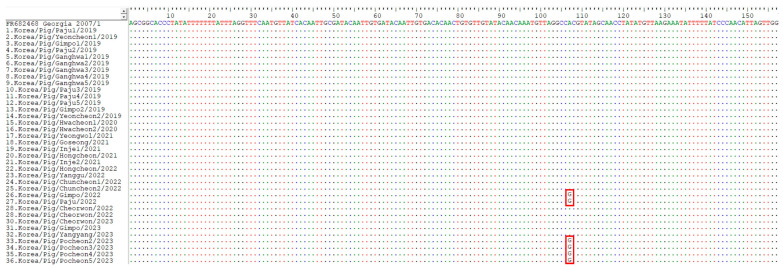
Analysis of IGRs between *A179L* and *A137R* (IGR_A179L-A137R_) of the 36 ASFV strains isolated from pig farms during 2019–April 2023. The ASFV strains from the 26th, 27th, 33rd, 34th, 35th, and 36th pig premises had a substitution of a single nucleotide from A to G at position 107 from the start of IGR_A179L-A137R_, corresponding to position 55405 in Georgia 2007/1.

The IGRs between *A179L* and *A137R* (IGR_A179L-A137R_) and *MGF 505 9R* and *10R* (IGR_MGF 505 9R/10R_), and the ECO2 region consisting of the IGR between *I329L* and *I215L* and the partial *I215L* gene, can also be used to trace the source of the virus [21,36,38]. One ASFV strain (ASFV/VN/Pig/Hanoi/07) reported in Vietnam was IGR_A179L-A137R_ I, which possessed one unit of an 11 nts TRS (GATACAATTGT) in IGR_A179L-A137R_, compared with Georgia 2007/1 and other Eurasian strains, which contain two repetitions of the TRS [21]. The 15 Korean ASFV isolates analyzed in this study were subjected to two repetitions of the TRS. However, the ASFV strains from the six pig premises (26th, 27th, 33rd, 34th, 35th, and 36th) had an A-to-G substitution at position 107 from the start of IGR_A179L-A137R_, corresponding to position 55405, according to Georgia 2007/1 (Figure 3). The localities of the six pig farms where ASFV cases with SNPs in IGR_A179L-A137R_ were identified are indicated in Figure 2c.

IGR_MGF 505 9R/10R_ variants can be divided into three types depending on the insertion of a 17 nt TRS (GATAGTAGTTCAGTTAA) [23,38]. However, IGR_MGF 505 9R/10R_ variants have recently been subdivided into eight types. MGF-1, including Georgia 2007/1, is the most common type, comprising five repetitions of a 17 nts TRS (AGTAGTTCAGTTAAGAT) with the structure ABBCD located proximal to the 9R gene and six units of a 17 nts TRS (AGTTCATTTAAGTCAAT) with the structure EFGHHH near the 10R gene. MGF variants vary in the number and type of TRS. MGF-2 to MGF-8 variants have been detected in Eastern European countries, including Latvia, Poland, Russia, Lithuania, and Romania [16].

*O174L*, *K145R*, and *MGF 505-5R* can be used in molecular epidemiology in combination with other genetic markers. The *O174L* variants are divided into three types: variant I, 100% identical to Georgia 2007/1; variant I, with an SNP; and variant II, with a 14 nt TRS (CAGTAGTGATTTT) insertion. *O174L* variants I and II have been detected in Romania, Poland, and Germany. *K145R* and *MGF 505-5R* have two types of variants: variant I, which is 100% identical to the reference strain Georgia 2007/1; and variant II, containing SNPs C65167A in *K145R* and G38332A in *MGF 505-5R* [25]. Genetic analysis of these three genes with CVR, IGR_I73R-I329L_, and IGR_MGF 505 9R/10R_ was useful in elucidating the introduction of ASFV and its transmission among European countries [16]. None of the 15 Korean isolates analyzed in this study contained variations in these three genes.

IGR_I329L-I215L_ and the partial *I215L* genes, named the ECO2 region, have recently been used to trace the origin of the virus in Eastern Europe. There are four ECO2 variants: the ECO2-I variant, with 100% sequence homology to Georgia 2007/1; ECO2-II, with an SNP at the 62nd position in *I215L*; ECO2-III, with an SNP in *A498G*; and ECO2-IV, with an SNP in *G466A*. Most ASFV strains in Europe and Asia were of the ECO2-I type, whereas the ECO2-II variants were detected in Romania, Bulgaria, Serbia, Greece, and North Macedonia between 2018 and 2022. The ECO2-III and ECO2-IV types have only been identified in China [16]. All 15 ASFV strains isolated from the pig farms in the present study were ECO2-I variants. *CP204L*, the *Bt/Sj* region, and the *J268L* region of the 15 Korean ASFV strains were identical to those of Georgia 2007/1, Chinese strains Pig/HLJ/2018 and China/2018/AnhuiXCGQ, and Vietnamese strain ASFV_NgheAn_2019, without variations.

All 15 ASFV strains isolated from the pig farms in South Korea between 2022 and April 2023 were p72 genotype II, serogroup 8, and CVR1. Fourteen ASFV isolates were IGR_I73R-I329L_ II, whereas the ASFV strain from the infected pig farm reported on 22 January 2023 was IGR_I73R-I329L_ I. Genetic analysis of the other seven genes indicated that the 15 strains were identical to the corresponding regions of the reference strain Georgia 2007/1. However, SNPs were detected in IGR_A179L-A137R_ in the ASFV strains from six pig farms. This suggests that there were other ASFV variants circulating in South Korea and the sources of the virus at the pig farms from which the IGR_I73R-I329L_ I and IGR_A179L-A137R_-SNP variants originated differed from those of other pig farms.

Investigations to elucidate the sources of these ASFV variants are underway. The IGR I variant was detected only at the 31st pig farm, which suggests that ASFV transmission from other pig farms can be ruled out. Contaminated pork products from affected foreign countries and domestic regions are possible, but since the first ASF case was confirmed on 17 September 2019, feeding with food waste at pig farms had been strictly prohibited and under the supervision of veterinary authorities. It is necessary to check whether the farm complied with the relevant laws. As the IGR I variant emerged in wild boars near the 31st farm in December 2019, wild boars are a strong potential source of the virus. However, the time difference between the identification of the IGR I variant in wild boars and the outbreak at the 31st pig farm was more than 3 years. Moreover, ASFV-infected wild boars had never been found in Gimpo City until April 2023. In this case, ecological changes, such as the migration of wild boars, which might have contributed to the spillover from wild boars to the pig farm, should be considered. With regard to the six pig farms where IGR_A179L-A137R_-SNP variants were detected, the two pig farms (26th and 27th) in Gimpo City and Paju City that reported cases on 28 September 2022 and four premises (33rd–36th) in Pocheon City that reported cases between March and April 2023 can be considered separately. The cases in the former two farms might have originated from the same source of infection, including wild boars. However, ASFV-infected wild boars had not been found in Paju City since April 2021. Ecological changes might have been involved in both outbreaks. The cases in the latter four farms, which could be clustered spatiotemporally, might have originated from the same source of infection. In Pocheon City, an ASFV-positive wild boar was detected in March 2023, which implies that wild boars may be a source of the virus there. More efforts, including consolidated surveillance of wild boars in the affected regions and investigations of potential ecological factors associated with wild boars, should be made to trace the sources of these ASFV variants.

Analysis of the whole-genome sequences of ASFV isolates is the gold standard in terms of molecular epidemiology, as it can enable the tracing of the source of the virus and extend our knowledge regarding virus evolution in South Korea. However, this method is time-consuming and may be restricted by the availability of information regarding the whole-genome sequences of comparable strains. In contrast, investigations of select gene markers of ASFV isolates using Sanger sequencing can be performed quickly to differentiate between the ASFV strains closely related to spatiotemporal patterns and share information with ease. Until the whole-genome sequence analysis is completed, the genetic variability within gene markers provides an opportunity to obtain deeper insights into the introduction and spread of ASFV in the affected country. Further studies should focus on attaining a better understanding of the ASFV subgroups circulating in South Korea by analyzing other potential gene markers and whole-genome sequences.

## Figures and Tables

**Table 1:** Genetic characterization results of the 36 ASFV isolates isolated from pig farms in South Korea between 2019 and April 2023.

Date of Outbreak		Isolate Name	Organ of Origin	qPCR C_t_	p72 Genotype	CD2v Serogroup	CVR	IGR_I73R-I329L_	IGR_A179L-A137R_	IGR_MGF 505 9R/10R_	ECO2	O174L	K145R	MGF 505-5R
1	16 September 2019	Korea/Pig/Paju1/2019	Spleen	17.1	II	8	CVR1	II	No deletion	MGF-1	ECO2-I	I	I	I
2	17 September 2019	Korea/Pig/Yeoncheon1/2019	Spleen	17.2	II	8	CVR1	II	No deletion	MGF-1	ECO2-I	I	I	I
3	23 September 2019	Korea/Pig/Gimpo1/2019	Blood	15.4	II	8	CVR1	II	No deletion	MGF-1	ECO2-I	I	I	I
4	23 September 2019	Korea/Pig/Paju2/2019	Spleen	15.3	II	8	CVR1	II	No deletion	MGF-1	ECO2-I	I	I	I
5	23 September 2019	Korea/Pig/Ganghwa1/2019	Blood	13.3	II	8	CVR1	II	No deletion	MGF-1	ECO2-I	I	I	I
6	25 September 2019	Korea/Pig/Ganghwa2/2019	Blood	15.4	II	8	CVR1	II	No deletion	MGF-1	ECO2-I	I	I	I
7	25 September 2019	Korea/Pig/Ganghwa3/2019	Blood	15.5	II	8	CVR1	II	No deletion	MGF-1	ECO2-I	I	I	I
8	26 September 2019	Korea/Pig/Ganghwa4/2019	Blood	16.0	II	8	CVR1	II	No deletion	MGF-1	ECO2-I	I	I	I
9	26 September 2019	Korea/Pig/Ganghwa5/2019	Spleen	17.6	II	8	CVR1	II	No deletion	MGF-1	ECO2-I	I	I	I
10	1 October 2019	Korea/Pig/Paju3/2019	Spleen	18.1	II	8	CVR1	II	No deletion	MGF-1	ECO2-I	I	I	I
11	1 October 2019	Korea/Pig/Paju4/2019	Blood	15.4	II	8	CVR1	II	No deletion	MGF-1	ECO2-I	I	I	I
12	2 October 2019	Korea/Pig/Paju5/2019	Spleen	16.4	II	8	CVR1	II	No deletion	MGF-1	ECO2-I	I	I	I
13	2 October 2019	Korea/Pig/Gimpo2/2019	Spleen	18.1	II	8	CVR1	II	No deletion	MGF-1	ECO2-I	I	I	I
14	9 October 2019	Korea/Pig/Yeoncheon2/2019	Blood	15.5	II	8	CVR1	II	No deletion	MGF-1	ECO2-I	I	I	I
15	8 October 2020	Korea/Pig/Hwacheon1/2020	Spleen	17.2	II	8	CVR1	II	No deletion	MGF-1	ECO2-I	I	I	I
16	9 October 2020	Korea/Pig/Hwacheon2/2020	Blood	34.3	II	8	CVR1	II	No deletion	MGF-1	ECO2-I	I	I	I
17	4 May 2021	Korea/Pig/Yeongwol/2021	Spleen	17.3	II	8	CVR1	II	No deletion	MGF-1	ECO2-I	I	I	I
18	7 August 2021	Korea/Pig/Goseong/2021	Spleen	17.1	II	8	CVR1	II	No deletion	MGF-1	ECO2-I	I	I	I
19	15 August 2021	Korea/Pig/Inje1/2021	Blood	13.2	II	8	CVR1	II	No deletion	MGF-1	ECO2-I	I	I	I
20	25 August 2021	Korea/Pig/Hongcheon/2021	Blood	25.7	II	8	CVR1	II	No deletion	MGF-1	ECO2-I	I	I	I
21	5 October 2021	Korea/Pig/Inje2/2021	Spleen	17.7	II	8	CVR1	II	No deletion	MGF-1	ECO2-I	I	I	I
22	26 May 2022	Korea/Pig/Hongcheon/2022	Spleen	20.8	II	8	CVR1	II	No deletion	MGF-1	ECO2-I	I	I	I
23	18 August 2022	Korea/Pig/Yanggu/2022	Spleen	19.8	II	8	CVR1	II	No deletion	MGF-1	ECO2-I	I	I	I
24	18 September 2022	Korea/Pig/Chuncheon1/2022	Spleen	15.1	II	8	CVR1	II	No deletion	MGF-1	ECO2-I	I	I	I
25	19 September 2022	Korea/Pig/Chuncheon2/2022	Blood	17.2	II	8	CVR1	II	No deletion	MGF-1	ECO2-I	I	I	I
26	28 September 2022	Korea/Pig/Gimpo/2022	Blood	15.8	II	8	CVR1	II	No deletion with SNP	MGF-1	ECO2-I	I	I	I
27	28 September 2022	Korea/Pig/Paju/2022	Lymph node	16.5	II	8	CVR1	II	No deletion with SNP	MGF-1	ECO2-I	I	I	I
28	9 November 2022	Korea/Pig/Cheorwon/2022	Spleen	17.9	II	8	CVR1	II	No deletion	MGF-1	ECO2-I	I	I	I
29	5 January 2023	Korea/Pig/Pocheon1/2023	Blood	15.0	II	8	CVR1	II	No deletion	MGF-1	ECO2-I	I	I	I
30	11 January 2023	Korea/Pig/Cheorwon/2023	Blood	19.3	II	8	CVR1	II	No deletion	MGF-1	ECO2-I	I	I	I
31	22 January 2023	Korea/Pig/Gimpo/2023	Spleen	20.1	II	8	CVR1	I	No deletion	MGF-1	ECO2-I	I	I	I
32	11 February 2023	Korea/Pig/Yangyang/2023	Spleen	16.5	II	8	CVR1	II	No deletion	MGF-1	ECO2-I	I	I	I
33	19 March 2023	Korea/Pig/Pocheon2/2023	Spleen	18.1	II	8	CVR1	II	No deletion with SNP	MGF-1	ECO2-I	I	I	I
34	29 March 2023	Korea/Pig/Pocheon3/2023	Spleen	19.1	II	8	CVR1	II	No deletion with SNP	MGF-1	ECO2-I	I	I	I
35	31 March 2023	Korea/Pig/Pocheon4/2023	Blood	21.3	II	8	CVR1	II	No deletion with SNP	MGF-1	ECO2-I	I	I	I
36	13 April 2023	Korea/Pig/Pocheon5/2023	Blood	22.0	II	8	CVR1	II	No deletion with SNP	MGF-1	ECO2-I	I	I	I

**Table 2:** Comparison of tandem repeat sequences (TRS) in the intergenic region (IGR) between *I73R* and *I329L*.

GenBank	Strain	Country	Date of Collection	Origin	IGR_I73R-I329L_	Reference
FR682485	Georgia 2007/1	Georgia	April 2007	Domestic pig	I	[37]
OQ030809	Pol17/WB/CASE237	Poland	13 March 2017	Wild boar	I	[16]
MK189457	China/Jilin/2018/boar	China	16 November 2018	Wild boar	I	[33]
MZ812370	VNUA Hanoi-ASF2	Vietnam	24 September 2019	Domestic pig	I	[35]
MT300324	Korea/19S3965wb/2019	South Korea	3 December 2019	Wild boar	I	[31]
OQ417675	Korea/Pig/Gimpo/2023	South Korea	22 January 2023	Domestic pig	I	This study
KJ627206	Pol14/Sz	Poland	14 February 2014	Wild boar	II	[20]
MH717104	ASFV-SY18	China	July 2018	Domestic pig	II	[5]
MN603969	Korea/Pig/Paju1/2019	South Korea	16 September 2019	Domestic pig	II	[6]
MN817979	Korea/19S804/wb/2019	South Korea	2 October 2019	Wild boar	II	[8]
MZ812354	VNUA QNinh-ASF2	Vietnam	2 May 2019	Domestic pig	II	[35]
OQ030832	POL17/DP/OUT66	Poland	3 August 2017	Domestic pig	III	[16]
MK670729	China/Guangxi/2019/Domestic pig	China	7 March 2019	Domestic pig	III	[34]
MT300325	Korea/19S5464/wb/2019	South Korea	30 December 2019	Wild boar	III	[31]
MZ812411	VNUA BG-ASF3	Vietnam	22 June 2020	Domestic pig	III	[35]
MZ812475	VNUA BG Hanoi-ASF9	Vietnam	7 August 2021	Domestic pig	III	[35]
OQ030919	POL18/WBCASE3314	Poland	13 December 2018	Wild boar	IV	[16]
MT889536	Pol19_01529_C88/19	Poland	2019	Wild boar	IV	[25]
MT889539	Pol19_04468_O1/19	Poland	2019	Domestic pig	IV	[25]
MT951774	Pol20_32983_O15/20	Poland	2020	Domestic pig	IV	[25]
ON053211	VNUA HB-ASF2	Vietnam	19 October 2021	Domestic pig	IV	[36]
ON053216	VNUA VP-ASF7	Vietnam	3 November 2021	Domestic pig	IV	[36]

## Data Availability

The data presented in the study have been deposited in GenBank under the accession numbers OQ417675~OQ417695 and OQ948243~OQ948252. The other data from this study are available on request from the corresponding author. The original contributions generated for the study are included in the article. Further inquiries can be directed to the corresponding author.
